# Mild Magnetic Hyperthermia Stimulation Attenuates b‐Amyloid Deposition in the Hippocampus of Alzheimer’s Disease Mouse Model

**DOI:** 10.1002/alz.088468

**Published:** 2025-01-03

**Authors:** Byeong Tak Jeon, Min‐Ho Kim

**Affiliations:** ^1^ Kent State University, Kent, OH USA; ^2^ School of Biomedical Sciences, Kent State University, Kent, OH USA

## Abstract

**Background:**

Accumulation of β‐amyloid (Aβ) plaque in the brain is a pathological hallmark of Alzheimer’s Disease (AD). We recently reported that the application of mild magnetic hyperthermia is feasible to target and disrupt Aβ plaques by means of generating localized heat on the surface of magnetic nanoparticles (MNPs) targeted to Aβ aggregates in response to a remotely applied alternating magnetic field (AMF) (Nanomedicine:NBM, 2021). The objective of the current study is to demonstrate the feasibility of mild magnetic hyperthermia stimulation (MNP/AMF) in clearing Aβ deposits *in vivo* using 5xFAD mice, a well‐established transgenic AD mouse model.

**Method:**

In this study, 5xFAD mice (9‐10 months old, males and females) were administered with MNPs (2 mg in 1 mL PBS, 20 nm size, Ocean Nanotech) or vehicle (1 mL PBS) via intracranial injection to the hippocampus using stereotaxic surgery. At day 7 post‐MNP injection, mice were stimulated with a single session of AMF (375kHz at 60 kA/m) for 15 min. At day 14 post‐MNP injection, brain samples containing the hippocampus were harvested and immunostained for Aβ burden (6E10 antibody) and microglial activation (Iba antibody). The number and size of Aβ plaques as well as changes in microglial morphology were assessed in the hippocampus area.

**Result:**

5xFAD mice treated with a single session of MNP/AMF showed enhanced clearance of Aβ plaques compared with the vehicle‐treated 5xFAD mice. The size of Aβ plaques in MNP/AMF‐treated mice decreased by 47.6% compared with the size in control mice. However, the number of Aβ plaques was not significantly reduced. MNP/AMF‐treated mice revealed an increased number of microglia in the proximity of Aβ plaques in the hippocampus by 37.8% compared with control mice. Additionally, the coverage of microglia in Aβ plaques increased by 44.8% under the MNP/AMF condition. Interestingly, this change was associated with a transition of microglia morphology from the ramified‐like into the phagocytic ameboid‐like shape in MNP/AMF‐treated mice, suggesting that MNP/AMF stimulation might skew microglial activation towards enhanced clearance.

**Conclusion:**

Collectively, our findings suggest that MNP/AMF stimulation could not only mediate the fragmentation of Aβ plaques but also activate microglia to clear Aβ.

